# Traditional herbal medicine legislative and regulatory framework: a cross-sectional quantitative study and archival review perspectives

**DOI:** 10.3389/fphar.2025.1475297

**Published:** 2025-01-30

**Authors:** Sileshi Dubale, Rashed Edris Usure, Yesuneh Tefera Mekasha, Gemmechu Hasen, Firdos Hafiz, Dereje Kebebe, Sultan Suleman

**Affiliations:** ^1^ School of Pharmacy, Mattu University, Mattu, Ethiopia; ^2^ School of Pharmacy, Department of Pharmaceutical Chemistry, Hawassa University, Hawassa, Ethiopia; ^3^ Pharmaceutical Sciences, Pharmaceutical Quality Assurance and Regulatory Affairs, University of Gondar, Gondar, Ethiopia; ^4^ Jimma University Laboratory of Drug Quality (JuLaDQ), School of Pharmacy, Institute of Health, Jimma University, Jimma, Ethiopia; ^5^ Medicine Registration and Licensing, Ethiopian Food and Drug Administration, Addis Ababa, Ethiopia; ^6^ School of Pharmacy, Pharmaceutics, Jimma University, Jimma, Ethiopia

**Keywords:** cross-sectional study, archival review, legislative, regulatory framework, traditional herbal medicine, Ethiopia

## Abstract

**Background:**

The World Health Organization (WHO) reports that a significant portion of the global population relies on traditional herbal medicine (THM) due to limited access to safe and high-quality modern medical care. In developing countries, it is difficult to guarantee the safety and quality of THM due to weak enforcement of the legal and regulatory framework. Hence, the study attempted to evaluate the country’s legislative and regulatory framework by comparing it with developed and developing countries that have well-established systems and identify gaps for future roadmaps in the THM landscape.

**Methods:**

A cross-sectional study and archival review were performed from November 2021 to March 2022 G.C. to contrast the legislative and regulatory framework for THM regulation with other selected countries like Africa, India, and China. A total of 237 regulatory personnel participated in the study. Data were collected through an archive assessment, self-administrative questionnaires, and literature searches. Secondary data were extracted from the archival review, and the findings were summarized and presented in tabular and text formats. The quantitative data were analyzed using Statistical Package for the Social Sciences (SPSS) software version 26, with outputs presented in text, table, and figure form.

**Results:**

The archival review of the study found that Ethiopia’s THM legislative and regulatory framework is still in a developmental phase, particularly when compared with countries that have more established systems. A cross-sectional study indicated that approximately 79.7% of participants were aware of THM-related content in the current legislation. However, 82.3% reported they had not received any formal training on THM regulations. For future roadmaps, 73.8% of respondents believed the government showed a commitment to supporting THM regulation, though 51.9% of participants noted limited knowledge and awareness of THM practices and product regulations. In terms of quality, safety, efficacy, rational use, and storage conditions, 49.8% of respondents rated regulatory implementation practice as not satisfactory. In this study, most study participants raised concerns about the performance of quality control parameters. Among regulatory experts, weak performance was identified in the practical implementation of THM regulatory activities, with 70.2% of weak performance observed at the federal level and 41.7% at the regional level. Key barriers to effective regulation included a lack of research on herbal medicines (90.3%) and insufficient regulatory mechanisms (87.8%). Additional challenges for regulatory offices included traditional healers’ reluctance to engage with scientific communities (56.5%), inadequate inspections (55.3%), and limited data on the safety, quality, and efficacy of certain medicinal plants (54.4%).

**Conclusion:**

Overall, the Ethiopian Food and Drug Authority (EFDA) is significantly strengthening the legislative and regulatory framework for traditional herbal medicines (THM), although full implementation is still forthcoming. This study highlights the need for comprehensive policy development, improved training initiatives, and reinforced regulatory systems to effectively monitor and regulate THM practices. For future roadmaps, collaboration among traditional healers, regulatory bodies, and scientific communities, along with supporting evidence-based research, could further enhance THM regulation in Ethiopia. These collaborative endeavors are critical for promoting the safety and quality of products derived from herbal medicines.

## 1 Introduction

The World Health Organization (WHO) estimated that about one-third (1/3) of the global population lacks access to conventional medicine and the opportunity to avail themselves of modern healthcare services (Oyebode et al., 2016). Hence, much of the world population uses traditional medicines (TMs) to fulfill their healthcare needs (WHO, 2019; WHO, 2013). A report from the World Health Organization revealed that around 80% of individuals in developing nations rely on herbal medicine for their primary healthcare needs (WHO, 2013). However, the presence of inadequate legal herbal frameworks (Usure et al., 2024) has hindered the herbal medicine sectors. The existing body of literature indicates that modern drug regulation systems receive significant attention, while traditional governance practices, which are largely unrecorded, are often disregarded (Chebii et al., 2020).

The high occurrence of antimicrobial resistance in sub-Saharan African nations (Mekasha and Godena, 2021) necessitates a shift away from modern medications toward utilizing locally sourced herbal remedies. Herbal remedies are cost-effective, readily accessible, less prone to resistance, and affordable (Mahomoodally, 2013; Thakur et al., 2011). The rise in the usage of herbal-based products throughout the region has led to growing concerns regarding the quality and safety of these products, which in turn may have implications for public health (Ara et al., 2020; Barkat et al., 2021). A well-organized, sustainable governance system is essential for ensuring a consistent supply of traditional medicine. This can be achieved by implementing sustainable practices such as conservation, cultivation, proper harvesting, regulated trade, and controlled use (Bussmann et al., 2016). The regulatory frameworks for herbal medicine in many African nations are currently inadequate and underdeveloped (Chebii et al., 2020; Usure et al., 2024).

As the demand for herbal medicinal products increases worldwide and numerous new products are introduced to the market, the importance of strong regulatory activity becomes evident. This is crucial in addressing public health concerns and ensuring the safety of these products. Although certain herbal medicines show promising potential and enjoy widespread usage, a significant number of them remain untested, and their usage is not adequately monitored (Moreira et al., 2014). This limits knowledge of their potential adverse effects and makes the identification of the safest and most effective therapies, as well as the promotion of their rational use, more difficult (WHO, 2002). It is also common knowledge that the safety of most herbal products is further compromised by a lack of suitable quality controls, inadequate labeling, and the absence of appropriate patient information (Raynor et al., 2011). In many regions of Africa, herbal remedies are commonly available for purchase in open markets, stores, and through traditional healers, as there is often a weak legal framework in place that does not require scientific evidence of their safety, effectiveness, or quality (Sharad et al., 2011).

Studies in sub-Saharan African countries such as Nigeria, South Africa, Ghana, and Uganda reveal significant gaps in policy implementation and design (James et al., 2018). Notably, Kenya lacks a registration system for herbal medicines, leading to unrestricted sales (Onyambu et al., 2019). In contrast to developing countries, thorough literature reviews conducted by regulatory bodies in various developed countries have played a significant role in formulating guiding principles to tackle issues concerning herbal medicines (Ramakrishna and Reddy, 2017). The legal status and utilization of herbal drug products differ greatly from country to country. Regulations in developed nations are considered to be the most thorough compared to other global regulations for herbal medicinal products (Lee et al., 2021). For instance, the United Arab Emirates (UAE) has acknowledged the importance of herbal medicines through federal law No. 20 of 1995. This legislation, titled “Medicines and products derived from natural sources,” specifically addresses the regulation of herbal medicines at a national level (Mirzaeian et al., 2019; WHO, 2019).

### 1.1 Archival review of legislative and regulatory framework of herbal medicine in selected developed and developing countries

The regulatory framework for herbal medicine is robust in economically advanced nations like the United States of America (United States), Germany, Japan, and the European Union (EU). For instance, the authorities that regulate HM in Germany, the United States, and Japan also regulate conventional medicines (WHO, 2019). In Germany, herbal medicines (HMs) are also regulated by the European Medicines Agency (EMA) due to Germany’s membership in the European Union (Peschel, 2007). The Traditional Herbal Medicinal Products Directive (THMPD), also known as Directive 2004/24/EC, amends Directive 2001/83/EC specifically for traditional herbal medicinal products. This directive allows herbal medicinal products to be sold with combinations of certain specified minerals and vitamins. These herbal products are readily available over-the-counter (OTC) to the general public in standard formulations, either as single herbs (simples) or in mixtures (Peschel, 2007).

The pharmaceutical medicine regulatory system in the United States is widely recognized as the global benchmark for ensuring the safety and effectiveness of drugs. Nevertheless, significant concerns have been raised regarding the adequacy of the regulation of herbal medicines (HMs) as dietary supplements in the country. The United States is the primary market for the pharmaceutical industry and currently offers around 20,000 HMs, valued at approximately US$ 62 billion. The World Health Organization predicts that this figure will soar to an astounding US$ 5 trillion by 2050 (Alostad et al., 2018). Hence, developed countries prioritize herbal medicines as equivalent to modern pharmaceuticals.

China, India, Pakistan, Brazil, and Bahrain are nations with extensive historical backgrounds and established practices of traditional medicine. These countries have taken significant strides in incorporating various herbal medicine products into their healthcare systems. This fact is supported by the substantial presence of HM products in both national pharmacopeias and national essential drug lists (NEDL) (Picking, 2024). Notably, China and India, as the leading producers and exporters of medicinal plant (MP) products, demonstrate commendable HM regulation among developing nations (He et al., 2015).

According to WHO reports, herbal medicines in India are regulated under the Ayurveda, Siddha, and Unani drugs provision in the Drugs and Cosmetics Act (WHO, 2019). Furthermore, herbal medicines (HM) are marketed in India with medical claims, health claims, and nutrient content claims, falling under the categories of prescription and nonprescription medicines. The Ayurveda, Unani, and Siddha pharmacopeias are recognized and enforced by law in India. Additionally, the Indian herbal pharmacopeia is utilized, although it is not held as legally binding. In the Indian market, prescription HMs are distributed in pharmacies, while nonprescription HMs, for self-medication or over-the-counter use, are available in pharmacies, other retail outlets, and through licensed practitioners (WHO, 2019).

In Bahrain, herbal medicines are sold as prescription medicines, nonprescription medicines, or OTC medicines in pharmacies and other outlets, in special outlets such as in HM stores, and by licensed practitioners (WHO, 2019; Alostad et al., 2019). The nation implemented regulations for herbal medicines that mirror those of traditional pharmaceuticals. In Bahrain, herbal medicines are categorized as prescription drugs, over-the-counter medications, herbal remedies, dietary supplements, and health products. Manufacturers of herbal medicines must adhere to the same manufacturing standards as those for traditional pharmaceuticals. To ensure compliance, manufacturers are obligated to provide samples of their products to a government-approved laboratory for testing and submit the latest good manufacturing practices (GMP) certification from their local authority. Safety requirements for herbal medicines in Bahrain are akin to those for traditional pharmaceuticals. All complementary and alternative medicine products (excluding herbal medicines specifically) must be registered in Bahrain (Alostad et al., 2019).

The WHO reported that Pakistan introduced the “Alternate Medicine Health Products Enlistment Rules” in 2014 to regulate HM (WHO, 2019). Herbal medicines are sold with claims about medical, health, and nutrient content, but these are unregulated in Pakistan. The American herbal pharmacopeia, WHO monographs, and EU monographs are used, but the information in the pharmacopeias is not legally binding. There are exclusive regulations for GMP, separate from those for conventional pharmaceuticals. The mechanisms that ensure compliance and t regulatory acts to control manufacturing have yet to be enacted in Pakistan (WHO, 2019). Traditional use without demonstrated harmful effects is considered sufficient for the safety assessment of HMs. There are no restrictions on selling herbal products. The annual market sales for HM in Pakistan in the years 2007, 2008, and 2009 were estimated by the Pakistan Tibbi and Homeopathic Manufacturers Association data to be US$ 5.5 million, US$ 6.5 million, and US$ 7 million, respectively (WHO, 2019; Rasheed et al., 2019).

The 2019 WHO survey report indicates that only 23 countries out of 54 African countries sales herbal medicine as per claims as per regulatory guidelines, while countries such as Eretria and Tunisia have no reported claims related to herbal medicine sales. Information regarding this matter is not clearly available for other countries. Sao Tome, Principe, and Burundi are among the countries that sell herbal medicine with claims yet lack legal regulations for those sales (WHO, 2019). Research carried out in Nigeria revealed that among 16 key informants, 68.8% acknowledged the existence of a national policy on traditional medicine (TM), while 31.2% expressed disagreement on this matter. Additionally, 75% of the respondents confirmed that the enforcement of manufacturing standards for herbal medicines is guaranteed through regulatory measures, whereas 25% held a contrary opinion (Awodele et al., 2014). Only 25% indicated that licensed practitioners are involved in the sale of herbal medicines, while 75% believed that non-licensed practitioners are responsible for this (Awodele et al., 2014). In addition, 87.5% emphasized the necessity of support from the WHO in the form of workshops aimed at enhancing national capacity for monitoring the safety of herbal medicines.

A study conducted in Kenya (64) revealed that many unregulated herbal medicinal products had high levels of microbial contamination. The microbial loads in these unregistered samples ranged from 3.00 × 10^6^ cfu/mL to 1.56 × 10^10^ cfu/mL, which far exceeded the standards set by BP or USP, requiring levels below 100 cfu/mL. Among the microbial isolates, *E. coli* was found in approximately 75% of the unregistered product samples, followed by *Klebsiella pneumoniae*, *Enterobacter aerogenes*, and *S. aureus* in 70%, 60%, and 45% of the samples, respectively. *Salmonella* spp. was isolated in 40% of the samples, while *Shigella* spp. was found in 20% of the samples (Onyambu et al., 2013). These findings indicate that none of the unregistered samples met the microbial load limits set by pharmacopeias for both bacterial and fungal contamination. Therefore, it is vital to extend regulatory activities currently implemented for pharmaceutical medicines to herbal medicinal products in order to improve their microbial quality and safety.

African countries regulate HMs as herbal medicines, nonprescription medicines, prescription medicines, dietary supplements, health food, and functional food. In Africa, HMs are most often regulated in the herbal medicines category, followed by the nonprescription medicine, prescription medicine, and dietary supplement categories. However, some countries have multiple regulatory categories. Countries like Guinea, Mali, Mauritania, and Tanzania regulate them as herbal medicines, nonprescription medicines, and prescription medicines. In Mauritania, HM regulation has five different regulatory categories, whereas Nigeria regulates them as herbal medicines and different types of food (WHO, 2019; Kasilo et al., 2019).

The literature showed that the herbal medicine regulation systems in Kenya (Chebii et al., 2020), Ghana (Thakkar et al., 2020), and Uganda (Uganda NDA, 2020) provide valuable insights into the effectiveness of these countries’ approaches compared to Ethiopia. These countries have made significant strides in establishing regulatory frameworks that ensure the safety, quality, and efficacy of herbal medicines. They have implemented comprehensive legislation, established regulatory bodies, and developed guidelines for the registration and licensing of herbal products. In contrast, Ethiopia’s herbal medicine regulation system lacks such robust measures, leading to a lack of control over the quality and safety of herbal medicines in the country (Usure et al., 2024). This was why the authors became interested in the current context of comparing the legal framework of Ethiopian herbal medicine with that of a robust regulatory system for herbal medicine. Particularly in Ethiopia, there is no post-market surveillance system, restriction on the sale of herbal medicines, or guidelines for clinical trials using traditional medicines. An initiative has been made to establish guidelines for licensing and minimum standards for traditional practice and practitioners. A committee from the Ministry of Health (MoH), the Veterinary Drug and Animals Food Administration and Control Authority (VDFACA), and the Ethiopian Health and Nutrition Research Institute (EHNRI) is preparing standards for the safety, efficacy, and quality of traditional medicine. Different studies were conducted on the toxicology and efficacy of drugs in the Drug Research Department of the EHNRI (Teshome, 2017; Amare, 2010).

In Ethiopia, TM practiced by traditional healers constitutes the use of natural substances composed of plants, animals, and minerals as remedies, in addition to spiritual healing and some practices like bone setting. The Ethiopian flora is estimated to contain between 6,500 and 7,000 species of higher plants, of which about 12% are endemic, and more than 90% of TM preparations are of medicinal plant origin (James et al., 2018; Tesfahuneygn and Gebreegziabher, 2019). Despite the significance of HM uses, the safety and therapeutic value of HM products and practices cannot always be guaranteed and remains largely unregulated (Teshome, 2017; Abdisa, 2017; Abay, 2009). This study offers a comprehensive analysis of the regulatory framework for traditional herbal medicines (THM) in Ethiopia, comparing it to global standards. It highlights the risks associated with inadequate regulation, particularly the public health dangers posed by substandard or poor-quality herbal products. The finding underlines the critical need to strengthen regulatory enforcement to protect public safety and ensure the quality of THM. Specifically, the study evaluates the existing legislative and regulatory structures governing THM in Ethiopia, identifying areas for improvement that could align the country’s practices with internationally accepted standards.

## 2 Materials and methods

### 2.1 Study setting and period

The study was conducted from November 2021 to March 2022 in the selected region, namely Oromia and the Southern Nationalities, Nations, and Peoples (SNNPR) regions, and one administrative city of Ethiopia. Ethiopia, located at the horn of Africa, is one of the oldest civilizations and the second most populous country in Africa, with a diversity of cultures and languages (Crummey et al., 2024). Approximately 80% of the Ethiopian cultures support TM, of which 95% are sourced from plants (Abebe, 1986). Ethiopia operates under a federal government administration system, which is divided into 11 regional states and two city administrations. These divisions encompass diverse agro-ecological zones. Additionally, the country has a unified regulatory authority responsible for overseeing both food and drug regulations, as well as herbal medicine.

### 2.2 Study design

An institutional-based descriptive quantitative study and an archival review were conducted from November 2021 to March 2022 G.C. The archival review was employed to compare the legal framework for THM regulation in Ethiopia with that of various African countries, India, and China. The aim was to evaluate the comprehensiveness and adequacy of Ethiopia’s medical-legal basis. The selection of African countries was based on a thorough review that identified the regulatory status of THM in most African countries using a checklist (Supplementary File 1) that was developed based on WHO Conventional Medicine regulation data-gathering tools used in the second and third global surveys (WHO, 2019).

### 2.3 Source and study population

The source population for quantitative data was all federal and regional-level authorities, directorates, offices, and case teams that were directly involved in medicine and health regulation. For qualitative data, the source population was all federal and regional-level authorities, directorates, offices, and case teams that were selected for quantitative data, and other institutions and associations that work with health and medicine regulatory offices on THM regulation. The study population contained all selected federal and regional-level authorities, directorates, offices, and case teams that were directly involved in medicine and health regulation and executed THM. The randomly selected sample frames for the present study lists were found in Supplementary File 2.

### 2.4 Inclusion and exclusion criteria

The study included federal and regional-level authorities, directorates, offices, and case teams directly involved in medicine and health regulation, specifically those with regulatory experience and information on THM. Additionally, participants from selected offices, case teams, and associations were included, as well as traditional herbalists who had been registered and actively practicing for 2 years or more. Regulatory officials who were not engaged in THM regulation-related activities were excluded from the study. Furthermore, consumers of THM and members of patient advocacy organizations were also excluded.

### 2.5 Sample size determination

For the quantitative data, because the source population of this study was medicine and health regulatory offices (MHROs), the sample size of the study was determined by the total number of study units that existed in the selected MHROs. In view of that, EFDA has 113 regulatory personnel in its four selected directorates. The regional medicine and health regulatory offices regulated TM by two case teams (health facility control (THM facility) and product quality control). Accordingly, based on the standard human resource allocation of Oromia and SNNPR regions, health bureaus, the regional-level MHROs should contain 20 regulatory personnel, and each zonal- and town-level MHRO should contain four regulatory personnel. As a result, the total regulatory personnel working in selected MHROs of the Oromia region for quantitative data was estimated to be 84 (20 at regional offices + (4 × 7 at zones) + (4 × 9 at towns) = 84, while 60 regulatory personnel were estimated to work in selected MHROs of the SNNPR region [20 at regional offices + (4 × 5 at zones) + (4 × 5 at towns) = 60].

In addition, 128 regulatory personnel were estimated to work in selected MHROs of Addis Ababa city for quantitative data [20 at city-level Food, Medicine, and Healthcare Administration and Control Authority (FMHACA) + (4 × 6 at sub-cities) + (14 × 6 at woredas)]. Therefore, the total expected number of regulatory personnel working in MHROs selected from the federal and regional levels for quantitative data was 385 (113 in EFDA, 84 in Oromia, 60 in SNNPR, and 128 in Addis Ababa). As a result, because the study units were small in number, the sample size calculation was not conducted. However, for various reasons, all study units that were estimated based on the standard did not participate in the study. The first reason is that some selected regional medicine and health regulatory offices contain fewer than estimated regulatory professionals. As a result, the total study units that were actually working in selected regional MHROs during data collection were 333 (67 in Oromia, 46 in SNNPR, 107 in Addis Ababa, and 113 in EFDA). In addition, of 333 regulatory personnel, some (24 employees) only worked for a month in selected regulatory offices. In addition, some regulatory personnel were not present in their offices during data collection times for different reasons. Thus, 237 of 309 regulatory personnel who met the inclusion criteria actually participated in the quantitative data collection, with a respondent rate of 76.7%.

### 2.6 Data collection method and sampling procedure

The sample was collected using a combination of an archive assessment of Ethiopia’s legislative and regulatory framework, self-administered questionnaires, and literature searches. Each data collection technique was primarily based on the WHO national TM policy guidelines and other related publications (WHO, 2019; WHO, 2002; WHO, 2013; Wambebe, 2009; Heinrich, 2015; Alostad et al., 2019; Demeke et al., 2022).

The homogeneity of the legal basis and organization structure of THM-regulated institutions/offices are affected by government administration structures. Therefore, the researcher selected a sample frame randomly from stratified medicine and health regulatory offices based on the hierarchal level of regulatory offices and the responsibilities of TM regulatory activities. Accordingly, the registration and regulation of THM products are implemented by EFDA in collaboration with the Ethiopia Public Health Institute (EPHI) and other agencies found under the MoH at the federal level, and the regulation of THM (practices, practitioners, and premises) is enforced by RHRBs that are established under regional health bureaus at regional, zonal, sub-cities, towns, and woredas health office levels.

Accordingly, in the case of city administration, the THM practices and practitioner regulation are implemented by EFDA offices of woreda administration under the supervision of city and sub-city administration levels of FMHACA offices. However, in the case of other regional states, the health and health-related services and product quality control authority or offices (HHRSPQCA/O) established under the regional health bureau and health offices of the zonal, town, and woreda administration levels were responsible for enforcing THM practices and practitioners’ regulations and registration. Additionally, two case teams (the health facility control case team and the health and health-related product quality control case team) are established under each health-related service and product quality control (HHRSPQC) and FMHACA authority or office. Based on this, the researcher randomly selected a sample frame from a stratified target population of federal and regional health sector administration levels of medicine and health regulatory authorities, directorates, offices, and case teams.

For the quantitative study design, all eligible individuals within the selected authorities, directorates, offices, and case teams who met the inclusion criteria were included. Additionally, a purposive sampling method was used to select key informants (KIs) from registered THM practitioners actively practicing in the selected zones, towns, and woredas of the Oromia region and Addis Ababa city. This approach ensured that the sample represented both regulatory personnel and experienced THM practitioners relevant to the study’s focus.

Accordingly, a selected sample frame was composed; four directorates of EFDA were purposefully selected (product assessment and registration, licensing and inspection, product safety, and product quality assessment). Likewise, of a total of 11 regional health regulatory bodies (RHRBs), three (30%) RHRBs (Oromia, SNNPR, and Addis Ababa city) were randomly selected and included in the study.

Furthermore, 30% of zonal and town-level health regulatory office administrations of Oromia and SNNPR regions and 30% of sub-cities and woredas-level health regulatory offices of Addis Ababa city were randomly selected for the present study. Consequently, the researcher consisted of 12 Zonal (seven from Oromia and five from SNNPR) and 14 Zonal Town (nine from Oromia and five from SNNPR) administration HHRSPQC offices or case teams for the study. In addition, the FMHAC offices case teams of four sub-cities and 14 woredas of Addis Ababa city administration were involved.

The selection of African countries used for comparison considered the literature availability in English, THM regulation status, and geographical proximity of each selected country to Ethiopia. As a result, three African countries, Ghana, Uganda, and Kenya, which scored more than 90%, 75%, and 20% on the reviewed checklist, respectively, were selected (Supplementary File 3). India and China were selected purposely due to having good status on THMs regulations. The checklist used in the review process was revised and edited to make it suitable for evaluating the content of the reviewed countries’ legal bases on THM regulatory activities.

### 2.7 Study variables

#### 2.7.1 Independent variables

Awareness and belief of respondents on THM regulation; Opinion of regulatory personnel about HM quality, safety, efficacy, rational use, storage condition, and THM regulation performance of regulatory offices; THM practices and product regulation plans, monitoring and evaluation, awareness, reporting trend, and sanctions applied to THM-related problems; and Performance of regulatory offices on THM practical regulation were included under independent variables.

#### 2.7.2 Dependent variables

Policy, legislation, and regulation; system and structure for THM regulatory activities; and THM regulatory and implementation outcomes were included as dependent variables.

### 2.8 Data quality assurance

To ensure the quality and reliability of data collection tools, a thorough review and revision process was undertaken, focusing on the legal and practical aspects of THM regulation. This process included adapting tools to reflect the specific regulatory context of THM. Language experts translated the interview questionnaires for THM practitioners into Afan Oromo and Amharic, the local languages, to ensure understanding. These translations were then back-translated into English by different experts to verify consistency and accuracy. High-quality data collection was further ensured by using well-designed, refined, and pre-tested tools. During data collection, data controllers assigned codes to each respondent to ensure the confidentiality of the respondents and the organization. Six pharmaceutical quality assurance experts (SD, REU, YTM, GH, DK, and SS) and one expert from the medicine registration and licensing departments (FH) collaborated to validate the final tools, bringing their combined expertise to enhance data quality assurance. This rigorous validation process solidified the reliability and alignment of the tools with the study objectives, ultimately strengthening the credibility and validity of the research findings.

### 2.9 Data analysis and presentation

Regarding the archival review, the first secondary data of different legal bases of those selected countries were extracted into pre-prepared tables in Microsoft Word 10. Then, the extracted data were analyzed to determine the presence of legal provisions for selected key parameters. Finally, the archival review findings data were summarized and presented in tabulate and text format for each reviewed country in a descriptive way under the topic selected for the review. The quantitative data were analyzed by organizing the collected questionnaire data after checking for completeness, consistency, categorization, and coding. Then, the organized data were entered into Epi-data manager version 4.6. Data were exported and analyzed using Statistical Package for the Social Sciences (SPSS), version 26.0 software. The data were analyzed in descriptive statistics. Finally, the analyzed data outputs were presented in text, table, and figure form.

### 2.10 Ethical considerations

The researcher maintained awareness of ethical considerations during the study. Prior to starting data collection, the researcher obtained ethical clearance from the Institutional Review Board (IRB) of Jimma University with reference number JHRPG/912/2021G.C. and a letter of cooperation from the school pharmacy. Then, a formal letter of cooperation was received from the relevant administrative bodies of the institutions selected for study. In addition, official informed consent was requested from each study participant during data collection by explaining the objective of the study to respondents. Furthermore, the participants were encouraged as they participated voluntarily, ensuring that the information they provided was only used for the study purpose.

## 3 Results

### 3.1 Result summary of the archival review

Overviews of THM policy, proclamation and regulation, administration structure and regulation framework, TM product MA, THM practice and provider regulation, TM/THM education, and health insurance status in Ethiopia, Kenya, Uganda, Ghana, India, and China are presented in Tables 1–4. The results obtained from the archival review showed that the Ethiopian TM policy is integrated into the national drug policy of 1993, and Proclamation No. 1112/2019 of EFDA is a national law for TM products. Agencies and TM divisions under the MoH and Regional Health Regulatory bodies (RHR) bodies under regional health offices enforce TM regulation holistically with conventional medicine (CM) under one umbrella by the same allopathic professionals. TM/HM is defined as medicine in Proclamation No. 1112 on Article 2/9, whereas the definitions of TM/THM practice and practitioner are not addressed in this proclamation.

**TABLE 1:** Overview of current policies, laws, and regulations of selected countries on TMs.

Comparison features	Ethiopia	Kenya	Ghana	Uganda	India	China
National policy on TM/T&CM	The policy on TM was integrated into the National Drug Policy of 1993, and now an exclusive TM policy has been drafted	There are several national policy documents on TM, and now the Traditional and alternative medicine (T&AM) policy bill has been drafted	The Policy of Traditional Medicine Development in Ghana of 2005	The policy on T&CM is integrated with the National Medicines Policy of 2015	Various policy initiatives have contributed; the latest one is The National Health Policy of India in 2017	The Regulations of the People’s Republic of China on Traditional Chinese Medicine (TCM) of 2003 and the Opinions on Supporting and Promoting the Development of TCM of 2009
Current national laws on TM/T&CM of those selected countries	EFDA, Proclamation No.1112/2019, and now the Proclamation of “Ethiopian TM Administration” has been drafted	The Pharmacy and Poisons Act No. 5 of 2019, The Kenya Health Act of 2017, and TAMP Bill of 2019	A Ghana Public Health Act of 2012 and Ghana TM Practice Act of 2000	The Uganda National Drug Policy and Authority Act of 1993 and the Uganda T&CM Act of 2019	The Indian Medicine Central Council Act of 1970, the Homeopathy Central Council Act of 1973, and The Drugs and Cosmetics Act of 1940 amended in 2009	PRC Drug Administration Law of 2019, Law of the PRC on TCM of 2017, and The Law on Licensed Doctors of the PRC, commenced in 1998
Supreme legislation body of the country	Parliament	Parliament	Parliament	Parliament	Parliament	National People’s Congress
Regulation level of TM/HM&CM	At central, regional, and district medicine and health admin levels	At central and county govt medicine and health admin levels	At the central, regional, and district health admin levels	At the central and district local govt health admin levels	At central and state govt Ayurveda, Siddha, and Unani (ASU) Drug and AYUSH admin level	At central, state, and county TCM and health admin levels
Administrative bodies to enforce the law/regulation	EFDA, executive T&CM department under the MoH and HHRSPQC (A/D) or FMHACA office under regional and lower health admin offices	Pharmacy and Poisons Board, T&CM executive department under the MoH, and county-level health concerned offices	Food and Drug Administration (FDA) of Ghana, Traditional Medicine Practice (TMP) Council established under the MoH, Regional and District TMP Council and health offices	National Drug Authority of Uganda, National Council of T&CM practitioners, and district-level T&CM Council and health offices	At central and state govt ASU Drug Admin Department and IMCC under the Ministry of AYUSH and State govt admin level AYUSH division	Drug Regulatory Authority and TCM department under the State Council of the Central Govt, and the TCM department at county and above county government levels
Supervisory body	MoHs and regional health bureaus	MoH and county health executive departments	MoH of Ghana	MoH of Uganda	Ministry of AYUSH	State Council, National Health and Family Planning Commission and SATCM

Ethiopia has prepared a draft of exclusive TM policy and proclamation, guidelines for TM MA, and different TM product directives. HM products are regulated exclusively as a category of TM products with exclusive GMP and safety requirements. The only pathway for TM products is market authorizations (MAs), and there is as yet no registered TM product in Ethiopia. TM products are not included in the post-market surveillance system. Regulating TM products based on their claims and legally binding national pharmacopeia and monographs for HMs does not exist in Ethiopia. Furthermore, Ethiopia has legal provisions for TM products, market regulation, clinical trials, and promotion and advertising. Only indigenous TM practices and providers that are regulated under the regional level of the health regulatory division are available in Ethiopia. Currently, TM practices and providers are regulated by regional TM directives, and there is no national TM proclamation. Registration and licensing systems for TM healers are implemented in some regional states.

Some regional states do not have an advisory committee for TM practices and provider regulation. Regarding THM education, Ethiopia has no clear education policy for TM, and at university, only it provides a single course. In Ethiopia, there is no health insurance coverage for TM at all. The system of TM practices integration with the national healthcare system is now at a nascent stage. There is no means of encouraging the quality of THM practices other than periodic inspection. The summary results are presented in Tables 1–4.

### 3.2 Archival review of the legal basis of Ethiopia and five other countries

To evaluate the contents of the TM/HM law of Ethiopia, the study attempted to review the national medicine laws or acts of five selected countries (Kenya, Uganda, Ghana, India, and China) (Table 1). The review examined several key areas in TM/HM regulation, including policies, laws, and regulations specific to THM; the national framework for administering TM and THM; and the regulation of TM and herbal medicine (HM) products, as well as THM practices, providers, education, and health insurance-related legal foundations. The archival review revealed that Ethiopia’s TM policy was integrated into the National Drug Policy of 1993, with a dedicated TM policy drafted but not yet implemented. Enforcement of laws and regulations is primarily managed by the Ministry of Health (MoH) and the Health and Health-Related Service and Product Quality Control (HHRSPQC) or the Food, Medicine, and Healthcare Administration and Control Authority (FMHACA) through regional and lower-level health administration offices. In contrast, some other regions selected for comparison have shown strong initiatives to establish independent regulatory agencies for more robust enforcement of T/HM regulations.

### 3.3 National framework for TM/THM administration structure and regulation

As depicted in Table 2, there are herbal medicine governing bodies, including practitioners and practitioners. However, government and public research funding for THM &CM in most reviewed countries was not clear.

**TABLE 2:** National framework for TM/THM &CM administration and regulation in Ethiopia and selected countries.

Key characteristic	Ethiopia	Kenya	Ghana	Uganda	India	China
Definition of TM and CM practices in any current national T&CM law/act/proclamation	Only stated in a draft proclamation	As TM and alternative medicine	As TM and alternative medicine	As TM and complementary medicine	As AYUSH	As TCM
Definition of HM products in any current national medicine law/act/proclamation	As medicine	As health product	As HM products	As HM products	As Ayurveda, Siddha, and Unani (ASU) drugs	As Chinese HM and TCM decoction pieces
HM product governing bodies	EFDA and RHR bodies	Pharmacy and Poisons Board and County Govt health department	Herbal and Homeopathic Medicines (HHM) Department under the FDA of Ghana	National Drug Authority of Uganda	ASU Drug Admin departments of central and state govt levels	The Drug regulatory authority (DRA) of TCM division of state council and lower govt admin
T&CM practices and practitioners regulating bodies	At the national level by the MoH and by regional health regulators by allopathic professionals only	At the national and local govt levels by T&CM practitioners’ council and allopathic professionals	At national and state govt by TMP Council and allopathic professionals	At national and state govt by TMP Council and allopathic professionals	At central and state govt by the Central Council of Indian Medicine (CCIM) and the Division of AYUSH at the state admin by ISM Practitioners only	SATCM at the division and local govt level by TCM and allopathic professionals
Unity of command and regulation	Multiple agencies	Multiple agencies	Single	Single	Single	Multiple agencies
National programme on T&CM	Yes	Yes	Yes	Yes	Yes	Yes
National office on T&CM	Yes	Yes	Yes	Yes	Yes	Yes
National expert or advisory committee on T&CM	Yes	Yes	Yes	Yes	Yes	Yes
National research institute on T&CM	Yes	Yes	Yes	Yes	Yes	Yes
Government and public research funding for T&CM	Only govt fund	Not clear	No funding up to the end of 2016	Not clear	Both	Both
National plan for integrating T&CM into national health service delivery	Yes	Yes	Yes	Yes	Yes	Yes

In Ethiopia, herbal medicine (HM) products are defined as medicines under the current national medicine law/act/proclamation. However, in other countries referenced in Table 2, HM products fall under varied definitions. The archival review indicates that the policies, laws, and regulations in these countries regarding TM and THM cover definitions, product regulatory bodies, and the regulatory frameworks for practices and practitioners that reflect promising progress in legislative and regulatory landscapes. Additionally, the studied countries had established national plans to integrate HM products into their healthcare systems. Although these developments are encouraging, the review underlines the need for further efforts to fully develop and harmonize regulatory frameworks for THM across these regions.

### 3.4 Regulatory situation of TM/THM in Ethiopia and those selected countries

A comprehensive overview of the regulatory landscape surrounding imported herbal medicine (HM) products in various countries indicates the contrasting situation in Ethiopia. Although Kenya, Ghana, Uganda, India, and China have established clear legal regulations to govern the importation of HM products, Ethiopia’s regulatory framework remains ambiguous and needs further investigation based on evidence (Table 3). A notable disparity between Ethiopia and the countries mentioned in the study was the absence of a legal framework for post-market surveillance of safety concerning veterinary herbal medicine products in Ethiopia. The lack of a regulatory system for monitoring the safety and efficacy of imported herbal medicine products raises significant concerns regarding their quality and the potential for adverse effects. Without an effective mechanism to oversee these products post-market, there is an increased risk to public health, as unregulated herbal medicines may not meet necessary safety standards or could lead to harmful outcomes for consumers.

**TABLE 3:** Regulatory situation of herbal medicine (HM) products in Ethiopia and selected countries.

Key functions	Legal provisions	Ethiopia	Kenya	Ghana	Uganda	India	China
Law and regulation of TM/HM products	Current national law/act/proclamation and regulation/rules	Proclamation No.1112/2019	The Pharmacy and Poisons Act No. 5 of 2019	A Ghana Public Health Act of 2012	The UNDP and Authority Act of 1993 and The National Drug Policy and Authority (NDPA) Regulations of 2014	The Drugs and Cosmetics Act of 1940 and the Drugs and Cosmetics Act of 1940 and Rules 1945	The PRC Drug Administration Law of 2019
	Authorized national guidelines/directives for regulation or market authorizations (Mas)	No (only draft directives)	Yes	Yes	Yes	Yes	Yes
	TM/HM regulation type	Exclusive as a category of TM/HM products	Partly the same as Conventional Pharmaceuticals (CPs) as a category of herbal and complementary products	Partly the same as CPs as a category of HM products	Partly the same as CPs as a category of HM products	Under Ayurveda, Siddha, and Unani drugs, as HM products	Under TCM, as Chinese HM products and TCM decoction components
	Current status HM regulatory framework	At the draft and legislative stage	Implementation	Implementation	Implementation	Implementation	Implementation
Category of TM/HM	Category of TM/HM in the country	Yes	Yes	Yes	Yes	Yes	Yes
Categories of HM products for regulation or registration	Human HM products	Yes	Yes	Yes	Yes	Yes	Yes
	Veterinary HM products	No	Yes	Yes	Yes	Yes	Yes
	Finished products	Yes	Yes	Yes	Yes	Yes	Yes
	Crude HM products	No	No	No	Not clear	Not clear	Yes
	Locally manufactured HM products	Yes	Yes	Yes	Yes	Yes	Yes
	Imported HM products	Not clear	Yes	Yes	Yes	Yes	Yes
	Herbal cosmetics products	Yes	Yes	Yes	Yes	Yes	Yes
	Prescribed HM	Yes	Yes	Yes	No	Yes	Yes
	Non-prescribed HM	Yes	Yes	Yes	Yes	Yes	Yes
	Food supplement	Yes	Yes	Yes	Not clear	Yes	Yes
HM claims and regulations	HM claims and regulation type	No claim-based regulation	No claim-based regulation	Yes: medical, health, and nutrient content claims	No claim-based regulation	Yes: medical, health, and nutrient content claims	Yes: medical, health, and nutrient content claims
Pharmacopoeia and monographs	National pharmacopoeia for HM	No	No	Yes	No	Yes	Yes
	National monographs for HM	No	No	Yes	No	Yes	Yes
	Any other pharmacopoeia and monographs used in the country	Yes	Yes	Yes	Yes	Not clear	Not clear
Manufacturing regulation	Current GMP for TM/HM manufacturing	Exclusive GMP for TM/HM	Same GMP regulations as CP	Same GMP regulations as CP	Same GMP regulations as CP	Exclusive GMP regulations for Ayurveda, Unani, and Siddha (ASU) drugs	Same GMP regulations as CP and additional GAP as needed
	GMP guideline for TM/HM	Yes (drafted in 2021)	Yes	Yes	Yes	Yes	Yes
	Requirements to ensure manufacturing HM quality	Yes	Yes	Yes	Yes	Yes	Yes
	Mechanisms in place to ensure compliance with manufacturing requirements	Yes	Yes	Yes	Yes	Yes	Yes
Safety requirements for HM	Same safety requirements as for CP	No	No	No	Yes	No	Yes
	Exclusive safety requirements for HM products	Yes	No	Yes	No	No	No
	Traditional use without demonstrated harmful effects is sufficient	Yes	Yes	No	Yes	Yes	Yes
	Reference safety data in documented scientific research on similar products	Yes	Yes	No	No	No	No
	Toxicological study reports	Yes	Yes	Yes	No	No	No
Registration of HM	Registration system for HM	Yes	Yes	Yes	Yes	Yes	Yes
	Registration directive/guideline	Yes (drafted)	Yes (implement)	Yes (implement)	Yes (implement)	Yes (implement)	Yes (implement)
	MA pathway or application form for TM/HM products	One pathway	Two pathways	Two pathways	Three pathways	One pathway	Two pathways
	Classification of HM for market authorizations (Mirzaeian et al.)	Yes, according to WHO Africa region, category	Yes, according to indications and product contents	Yes, according to indications and product contents	Yes, according to WHO Africa region, category	Yes, according to indications and product contents	Yes, according to indications and mode of production
	How many HMs are registered	Nil	Not clear	375 HM in 2016	Not clear	Not clear	In 2017, more than 60,000 HMs
Post-marketing Surveillance	Post-market surveillance system for the safety of HM	Nil, only for CM	Yes, includes HM products	Yes, includes HMs since 2000	Yes, includes HM	Yes, includes HM under AYUSH	Yes, includes TCM
Market regulation	Legal provision on the regulation of HM sales	Yes	Yes	Yes	Yes	Yes	Yes
Clinical trials regulation	Legal provision to regulate TM/HM clinical trials	Yes	Yes	Yes	Yes	Yes	Yes
Promotion and advertising regulation	Legal provision to regulate TM/HM promotion and advertising	Yes	Yes	Yes	Yes	Yes	Yes

Compared to Kenya, Uganda, Ghana, India, and China, Ethiopia’s implementation of legal provisions for imported HM products was notably inadequate. This suggests that Ethiopia may be lagging in terms of ensuring the safety and efficacy of these products, potentially putting consumers at risk. Further details on the remaining regulatory circumstances surrounding herbal medicines in Ethiopia are presented in Table 3. The study outlines additional gaps in the country’s regulatory framework, shedding light on areas that require improvement to ensure the safe and effective use of imported HM products (Table 3).

### 3.5 TM/HM and CM practice, providers, education, and health insurance regulatory framework

As can be seen in Table 4, the regulation of TM/HM providers and practices was solely governed by the draft current national law, act, or proclamation. However, Kenya, Ghana, Uganda, India, and China have implemented regulatory frameworks to oversee the regulation of herbal medicine. Additionally, there are no national regulations or directives but rather some regional- and state-level practice regulations for TM/HM providers in Ethiopia.

**TABLE 4:** Overview of T&CM practice, providers, education, and health insurance regulatory framework in Ethiopia and selected countries.

Key features	Regulated practices	Ethiopia	Kenya	Ghana	Uganda	India	China
T&CM practices and providers available in the country	Various indigenous T&CM practices and providers	Yes	Yes	Yes	Yes	Yes	Yes
	Various non-indigenous T&CM practices and providers	No	Not clear	Yes	Yes	Yes	Yes
TM/HM &CM providers and practices regulation	Current national law/act/proclamation governing T&CM	No, only draft proclamation	The Kenya Health Act of 2017, the Traditional Health Practitioners (THPs) Bill of 2014, and Traditional alternative health medicine practice (TAHP) Bill of 2019	The Ghana TM Practice Act of 2000 (ACT 575)	Uganda T&CM Act of 2019	The IMCC Act of 1970 and The Homeopathy Central Council Act of 1973	The Law on Licensed Doctors of the PRC took effect in 1998
	Current national- and/or state-level regulation/guidelines/directives for regulation	No national regulations or directives, rather at some regional or state levels	The Kenya Health Act of 2017, county-level T&CM law, and TAHP Bill (Draft)	The Ghana TM Practice Act of 2000 (ACT 575)	Uganda T&CM Act of 2019	The IMCC Act of 1970 and The Homeopathy Central Council Act of 1973	The Law on Licensed Doctors of the PRC took effect in 1998
	Current status of T&CM regulatory framework	Yes (draft and implementation stage) and lack of uniformity	Yes (draft and implementation stage) and lack of uniformity	Yes (Implementation)	Yes (Implementation)	Yes, (Implementation)	Yes (Implementation)
	Regulatory level of indigenous T&CM providers and practices	Regional states and lower admin-level health regulatory division	National and county-level established regulatory bodies and health departments	National and state-level health departments and/or traditional medicine practitioners councils (TMPCs)	National and state-level health departments and/or TMPCs	Central and state admin-level AYUSH regulatory divisions	Central and state-level TCM regulatory divisions
	Non-indigenous T&CM providers and practices regulatory level	National level	National level	National level	National level	Central level	Central level
	T&CM professionals affected by regulations	Only allopathic professionals	TMPC and allopathic professionals	TMPC and allopathic professionals	TMPC and allopathic professionals	Only Indian Medical Service (IMS) professionals	TCM and allopathic professionals
	Registration system for T&CM healers/apprentices	Yes	Yes	Yes	Yes	No	No
	Registration of certified T&CM practitioners	Yes	Yes	Yes	Yes	Yes	Yes
	License is needed to practice T&CM	Yes (encouraged by the government)	Yes (compulsory)	Yes (compulsory)	Yes (compulsory)	Yes (compulsory)	Yes (compulsory)
	Who issues licenses for T&CM practices	Under the MoH at the T&CM division, RHB, and District health offices	Under the MoH at the T&CM division and county health departments	Under the MoH and state-level TMPC division	Under the MoH and state-level TMPC division	CCIM and its state-level divisions under the Ministry of AYUSH	TCM division at the Central State Council and State Administration
	Expert or advisory committee for T&CM practices and providers regulation	At some regional state health bureaus	Yes	Yes	Yes	Yes	Yes
Education of T&CM providers regulation	T&CM education policy	Yes (draft stage)	Yes (draft stage)	Yes (implement)	Yes (implement)	Yes (implement)	Yes (implement)
	T&CM program provided at the university level	No	Not clear	Yes	Not clear	Yes	Yes
	Level of T&CM attainable at the university level	Course	Not clear	Bachelor degrees	Not clear	Bachelor, masters, PhDs, and clinical doctorate degrees	Bachelor’s, master’s, and PhD degrees
	Are any training programs for indigenous T&CM practitioners run by govt officials?	Yes	Yes	Yes	Yes	No	No
	Are any other non-indigenous T&CM training programs run by govt officials?	No	Not clear	Yes	Not clear	No	No
Health insurance coverage for T&CM	Is indigenous T&CM covered by health insurance in the country?	No	No	Yes	No	Yes	Yes
	Are other T&CM practices covered by health insurance?	No	No	Partially	No	Yes	Partially
	Type of available health insurance provider	None	None	No public, only govt and private organization	None	Both public and private health insurance	No public, govt, or private health insurance companies
	Which T&CM practices get health insurance coverage	None	None	Indigenous TM, HM, TCM, chiropractic, and naturopathy	None	All T&CM services	Indigenous TM (TCM), HM acupuncture, and osteopathy
Integration of T&CM practice into the healthcare system	System of T&CM practice integration with allopathic healthcare services	At an ideal stage	At the legislative stage	At implementation	At implementation	At implementation	At implementation
	T&CM providers practice level	In private T&CM facilities	In private T&CM facilities	In private T&CM facilities	In private T&CM facilities	In both private and public T&CM facilities	In both private and public T&CM facilities
	Type of integration proposed	Not clear	Some practices in a parallel manner by a referral system	Practices in a parallel manner and referral system	Some practices in a parallel manner and a referral system	Practices in a parallel manner and some clinical	Will be completely integrated with clinical practice by the year 2030
Quality of T&CM care	Means of encouraging quality of T&CM care other than periodic inspection	None	None	None	None	Good practitioners are listed as E-Porter Practitioners, ensure patient safety through drug research	Certified practitioners are titled as Distinguished Doctors and ensure patient safety through research
T&CM practitioners association	Organization type	NGOs	NGOs	NGOs	NGOs	NGOs	NGOs
	Registration	Compulsory	No info	Compulsory	Compulsory	Optional	Compulsory
	Collaboration with government	Relatively poor collaboration	No info	Work closely with the MoH and state health departments	Work closely with the MoH and state health departments	Poor collaboration with the Ministry of AYUSH and state-level AYUSH departments	Work closely with SATCM

### 3.6 Quantitative results from regulatory personnel

#### 3.6.1 Demographic characteristics of respondents

The study involved a total of 237 regulatory personnel (Supplementary File 4). Most participants were male individuals (75.5%), pharmacists (66.2%), and held first degrees (83.1%). The participants were from different regulatory offices, namely EFDA (57), Oromia RHR offices (58), SNNPR RHR offices (41), and Addis Ababa RHR offices (81). On average, the respondents had 4.1 ± 2 years of experience in regulation, and 139 (58.6%) had ≤4 years of experience.

#### 3.6.2 Survey on awareness and opinions regarding THM legislation and practices

The study involved 237 individuals, focusing on their awareness and opinions concerning traditional herbal medicine (THM) legislation and practices. Approximately 79.7% of participants demonstrated awareness of the THM-related content in the current legislation. Additionally, only 65 respondents (34.4%) believed that the legislative content on THM was comprehensive and adequate. Regarding public health concerns, a significant majority (81.0%) of participants believed that the current methods of preparation and practice of THM practitioners could lead to severe public health issues. Furthermore, about 81.8% of respondents were aware that THM practices and product regulations were intended to protect the public from health problems. The study found that 82.3% of participants reported not receiving any training on THM regulations. Moreover, regarding government commitment, most participants (73.8%) believed that the government showed strong commitment and support for regulating THM (Table 5).

**TABLE 5:** Awareness and belief of respondents on THM regulation, Ethiopia, 2022, (n = 237).

Awareness and belief of respondents on THM regulation	Yes		No	
	N	%	n	%
Awareness of respondents of THM legislation	189	79.7	48	20.3
Comprehensiveness and adequateness of THM legislation	65	34.4	124	65.6
Current THM practice can cause severe public health problems	192	81.0	45	19.0
THM regulation can protect the public from THM-related health problems	157	81.8	35	18.2
Respondent’s belief on THM regulated by biomedicine professionals	183	77.2	54	22.8
Respondent experiences when attending THM regulation-related training	42	17.7	195	82.3
Government commitment and support for THM regulation	175	73.8	62	26.2

**FIGURE 1:**
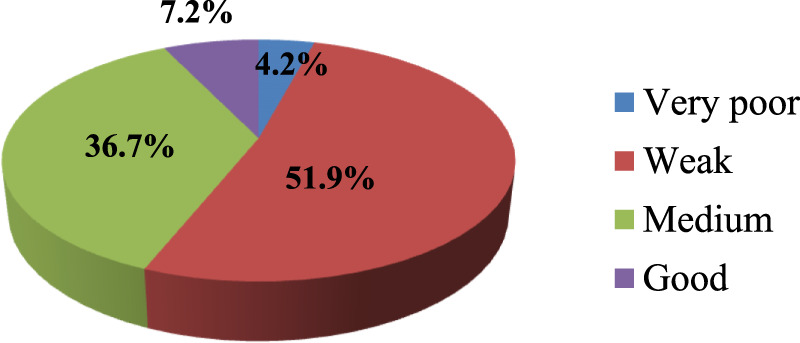
Percentage levels of respondents’ knowledge of THM practices and product preparation.

The study indicates an important finding about knowledge gaps in THM practices and preparation among participants. The finding revealed that around 51.9% of participants lacked sufficient knowledge, and 36.7% of respondents demonstrated medium knowledge. This distinction may imply that a significant portion of participants have limited understanding regarding herbal medicine practice and product preparation (Figure 1).

**FIGURE 2:**
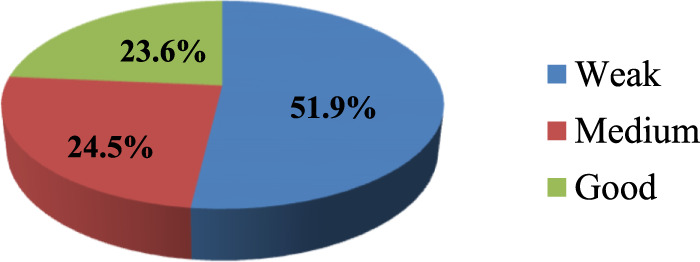
Respondents’ rate of awareness of THM regulation in Ethiopia.

As shown in Figure 2, about 51.9% of regulatory personnel had a weak awareness of herbal medicine regulation, suggesting significant gaps in knowledge and understanding. Only 24.5% exhibited a medium level of awareness, showing a partial understanding but not comprehensive familiarity with the regulations.

#### 3.6.3 Regulatory personnel on HM quality, safety, efficacy, rational use, and storage conditions of THM regulations

Of 237 study participants, most (57.8%, 51.9%, 54.0%, 58.2%, and 59.9%) respondents believed the traditional herbal medicine practice, safety, efficacy, quality, and HM rational uses in Ethiopia were not good, respectively (Table 6). The study’s findings revealed significant gaps in practices surrounding traditional herbal medicine (THM) among respondents, with only a small fraction demonstrating good quality control practices. An overall mean of 49.8% of respondents scored the system poorly in key areas such as safety, efficacy, quality, rational use, and storage conditions. These results indicate widespread concerns about the current standards in the THM market. Furthermore, the low percentage (6.8%) of respondents with good quality control practices underlines the urgent need for improved guidelines and educational initiatives to ensure that THM products meet safe and effective standards.

**TABLE 6:** Opinion of respondents on safety, efficacy, quality, rational use, and storage condition of THM currently available on the market, Ethiopia, 2022 (n = 237).

Variable	Very poor		Not good		Medium		Good	
	N	%	n	%	N	%	n	%
Safety of THM practices	17	7.2	137	57.8	72	30.4	11	4.6
Efficacy of HM preparation/products	34	14.3	123	51.9	66	27.8	14	5.9
Safety of HM preparation/products	28	11.8	128	54.0	77	32.5	4	1.7
Quality of HM preparation/products	46	19.4	138	58.2	53	22.4	0	0.0
Rational use of THM preparation/products	34	14.3	142	59.9	61	25.7	0	0.0
Quality of HM preparation methods used by registered THMPs	0	0.0	80	33.8	136	57.4	21	8.9
Storage condition of HM at the home of registered THMPs	0	0.0	79	33.3	96	40.5	62	26.2
Mean (%)	9.6%		49.8%		33.8%		6.8%	

#### 3.6.4 Regulatory performance of EFDA and regional health regulatory bodies on practical implementation of THM regulatory activities

**FIGURE 3:**
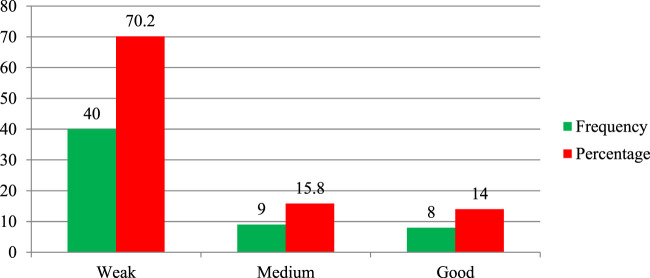
Performance of EFDA regulatory personnel (n = 57) on practical implementation of herbal product regulation.

**FIGURE 4:**
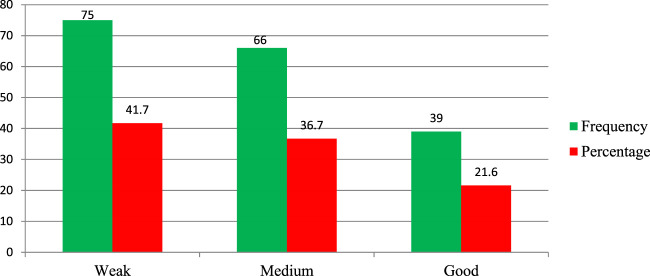
Performance of regional health regulatory bodies on the practical implementation of THM regulatory activities, Ethiopia (n = 180).

The study assesses the regulatory performance of personnel working in federal and regional offices, particularly focusing on their implementation of THM product regulation and practical implementation of regulatory activities. At the federal level, approximately 70.2% of the Ethiopian Food and Drug Administration (EFDA) regulatory personnel rated their authority’s overall performance in THM regulatory activities as weak (Figure 3). Only 14% of federal-level regulatory experts demonstrated good regulatory performance in handling herbal medicine product regulations. For regulatory experts working at regional health regulatory offices, the findings also reveal notable performance gaps. About 41.7% (75 of 180) of regional regulatory personnel reported weak performance in implementing THM regulatory activities, while only 21.6% of participants rated their implementation practices as good (Figure 4). These results underline significant gaps in the regulatory workforce’s effectiveness in managing THM product regulation, both at the federal and regional levels, and highlight areas in need of improvement for effective regulatory oversight.

#### 3.6.5 THM practices and product regulation plan, monitoring and evaluation

**FIGURE 5:**
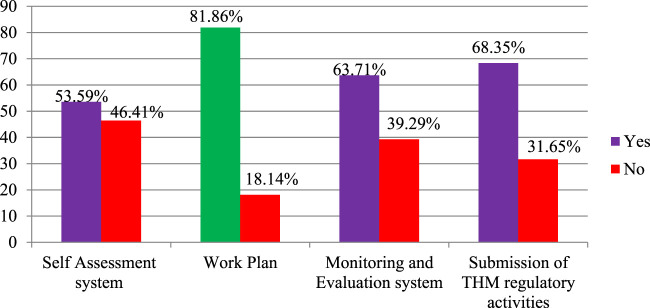
Plan, monitoring, and evaluation system for THM regulation, Ethiopia, 2022 (n = 237).

In this study, the regulation of THM practices was explored, with a focus on planning, monitoring, evaluation, and self-assessment processes within regulatory working environments. The findings indicated that 81.86% of participants agreed that a work plan exists for THM regulatory activities, ensuring a structured approach to regulating THM practices and products (Figure 5). Furthermore, most participants acknowledged the presence of monitoring and evaluation (63.7%) and reporting systems (68.4%) within THM regulatory activities, demonstrating foundational oversight mechanisms.

Self-assessment processes were also recognized, with 53.6% of participants agreeing on their existence. However, nearly half (46.41%) of the participants disagreed about the presence of a self-assessment system for THM practices and product regulation, highlighting a significant gap in internal evaluation processes that could impact the effectiveness of THM regulation.

#### 3.6.6 Awareness, reporting trends, and sanctions applied to THM-related problems

Most respondents who were aware of THM-related problems had not reported them to respective regulatory offices (Supplementary File 5). The Supplementary File 5 shows that unregistered THM services rendering premises and unregistered practitioners were reported by 18.6% (37/199) and 18.6% (40/215) of participants, respectively. More than 50% of those reported THM-related problems are receiving sanctions from regulatory offices. Most study participants noted that legal and administrative sanctions are applied to the reported problems. Most respondents reported that the absence of a formal reporting system, lack of strong legal branches on TM, supposing the problem is not severe for public health, lack of evidence to report the problems, lack of public support, weak legal enforcement, and educative legal and administrative sanctions not applied by regulatory offices are the reasons for not reporting THM problems.

#### 3.6.7 Factors affecting the performance of regulatory offices on the practice of THM regulation

**FIGURE 6:**
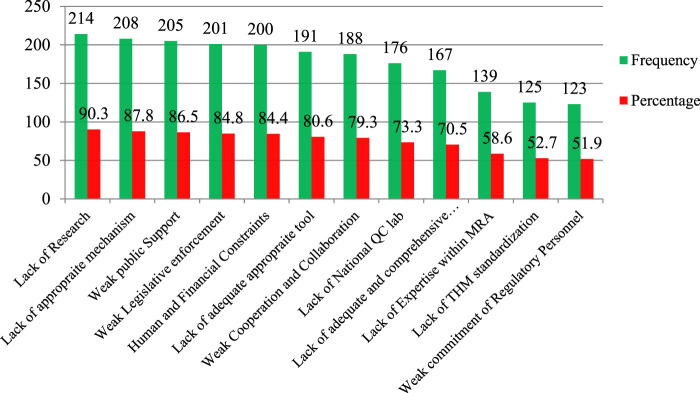
Factors affecting the regulation performance of medicine and health regulatory offices on THM practices, products, and practitioner regulatory implementation, Ethiopia, 2022 (n = 237). Note: QC Lab, quality control laboratory; MRA, Medicine regulatory authority.

Most respondents reported that a lack of research on HM (90.3%), a lack of appropriate mechanisms to regulate THM practice and products (87.8%), weak public support (86.5%), weak legislative enforcement (84.8%), human and financial resources constraints (84.4%), and inadequate basic THM regulatory tools (80.6%) (Figure 6) influenced the performance of the various regulatory offices.

#### 3.6.8 Challenges related to THM product, practice, and practitioner regulations

As can be seen in Table 7, respondents report that healers’ suspicions of and hesitancy to work with scientific communities (134, 56.5%), the inadequateness of current THM inspections using different regulatory tools (131, 55.3%), and the lack of scientific data for the safety, quality, and efficacy of some medicinal plants (129, 54.4%) were the major challenges of traditional herbal medicine products, practices, and provider regulation faced by the EFDA and regional health regulatory offices.

**TABLE 7:** Challenges of regulating THM products, practices, and providers faced by EFDA and RHR offices, Ethiopia, 2022 (n = 237).

Challenges related to THM products, practices, and providers regulation	n (%)
Healers are suspicious and hesitant to work with scientific communities	134 (56.5)
Inadequateness of current THM inspection using different regulatory tools	131 (55.3)
No scientific data on the safety, quality, and efficacy of some medicinal plant formulations	129 (54.4)
Lack of uniformity between regional TM directives and regulation	127 (53.6)
Lack of plan, monitoring, and evaluation on TM regulatory implementation	125 (52.7)
Healers are reluctant to accept even minor changes in HM practice and product	124 (52.3)
Lack of quantity and quality of the national and regional TM laboratory	122 (51.5)
Exclusive budget provision, human resources, and organization structure	117 (49.4)
Weak government support for zonal-, woreda- and town-level regulatory offices	113 (47.7)
Absence of mechanism to regulate the quality and safety of HM categories 1 and 2	112 (47.3)
Lack of healers’ knowledge of how TM products are registered	109 (46)
Government support for THM research and development is not sufficient	106 (44.7)
Regulatory personnel are viewed with suspicion by healers during inspection	101 (42.6)
Healers are superstitious about the safety and quality of THM practices and preparations	98 (41.4)
Lack of awareness of THM healers on THM registration and regulation	98 (41.4)
Lack of standardization HM product prepared by TM healers	88 (37.1)
Lack of attention and recognition of government and public for TM regulation	73 (30.8)
Healers superstitions about the efficacy, safety, and quality of HM products	72 (30.4)
Weak support for healers in promoting practice, skill, finance, and others	69 (29.1)
Lack of cooperation among enforcement organizations and offices	64 (27.0)
Training for healers on HM preparation, extraction, and dose adjustment	63 (26.6)
Lack of indigenous knowledge about conservation and documentation	57 (24.1)
Source THM knowledge is not based on written evidence and instead is secretive	56 (23.6)
Lack of interest and motivation of THM healers to be registered	53 (22.4)
Healers are registered based on testimonials about disease cures from communities	35 (14.8)
The federal government is more focused on HM product regulations and inadequately supports the regulation of THM practices and practitioners	24 (10.1)

According to the Ethiopian Food and Drug Authority and RHR personnel, there are several potential approaches to address the challenges in implementing and improving the regulation of herbal medicine. These include government support through the implementation of plans, strategies, and advocacies for traditional herbal medicine (THM), as well as providing training, financial resources, materials, and equipment for THM services. Additionally, it is important to establish formal clinical or service integration of traditional medicine (TM) within the national healthcare system and to offer formal, organized, supportive, and continuous training on THM practices. Furthermore, effective regulation should be implemented to control THM healers, and support should be provided for the establishment of strong national TM/THM associations and training institutions.

## 4 Discussion

The study aims to evaluate the legislative and regulatory framework of Ethiopian traditional herbal medicines using a facility-based cross-sectional study and an archival review. Herbal medicine has a long history of traditional use in Ethiopia, where various plants have been used to treat ailments for generations. Despite its historical roots and widespread use among communities, the recognition and regulation of herbal medicine by national authorities in Ethiopia are still in development (Tuasha et al., 2023). This lack of formal acknowledgment raises concerns about the safety and efficacy of these treatments (Usure et al., 2024; Demeke et al., 2022).

The existing evidence highlights several factors contributing to doubts about the quality, safety, and efficacy of traditional herbal medicine-based products, such as lack of standardization, limited scientific research, regulatory gaps, integration with modern medicine, and education and awareness (Eruaga et al., 2024; Noviana et al., 2022). Efforts to bridge these gaps include promoting research on herbal medicines, improving regulatory frameworks, and fostering collaboration between traditional healers and modern healthcare practitioners (Mbwambo et al., 2007; Van der Watt et al., 2017). These steps are critical to ensuring that the benefits of traditional herbal medicine can be safely and effectively integrated into the broader healthcare system in Ethiopia.

This comparative archival review examines the differences between Ethiopian traditional herbal medicine and other widely practiced regions such as Kenya, Uganda, Ghana, India, and China. The integration of Ethiopia’s TM/HM Policy into the national drug policy occurred in 1993, making TM products a part of the national legislation. Despite this, there are no registered TM products, no national pharmacopeia or monographs for HM products, and no defined education policy for TM (Usure et al., 2024). There is also no health insurance coverage for TM and limited collaboration between TM healers’ associations and regulatory authorities. This study highlights the varying stages of TM policy integration, regulatory frameworks, and education across the selected countries, with Ethiopia showing significant progress in drafting exclusive THM policies but still lagging in several areas, such as product registration, post-market surveillance, and education policy. Notable insights from other nations: Kenya and Uganda exhibit comparable integrated policies that encompass national frameworks for the administration and regulation of traditional medicine (UNESCO, 2021).

In contrast, Ghana demonstrates a more sophisticated integration of TM within its national healthcare system, supported by specialized regulatory authorities (Ampomah et al., 2023; WHO, 2014–2023). Furthermore, India and China possess thorough legal frameworks and extensive regulations governing TM products and practices, which include well-developed educational systems and health insurance provisions for TM (Fan et al., 2012; WHO, 2014–2023). The finding indicates a significant gap in the EFDA proclamation compared to the medicine acts of Ghana, Uganda, and Kenya. The review indicates that the medicine acts of these countries provide a more comprehensive definition of medicine based on its functions and sources and that they include detailed regulatory activities for traditional and complementary medicines (T&CMs), which might not be as explicitly outlined in the EFDA proclamation. Addressing these gaps could involve updating the EFDA proclamation to include clearer definitions and detailed regulatory frameworks for T&CMs, aligning more closely with the practices observed in Ghana, Uganda, and Kenya (Ghana FDA, 2012; NDA, 2021; PPB, 2011). This could enhance regulatory oversight and improve the quality and safety of medicines, including traditional and complementary types, in Ethiopia.

The study showed that around 79.7% of the participants were aware of THM legislation. Out of those who knew, only 65 respondents (34.4%) believed that the legislation on THM-related matters was comprehensive and adequate. This indicated that the existing regulations were not adequate. A similar report from Tanzania indicated that awareness of regulations and tools used for regulating the T&CM operations among practitioners was generally very low (Mujinja and Saronga, 2022). The respondents think that the current THM practices could lead to severe public health issues (81.0%). In a similar report from Uganda, most respondents were quite aware of the importance of TM in the sustenance of the healthcare system (Galabuzi et al., 2010). Additionally, 81.8% of the participants, which is about 157 of 192, were informed about the existence of regulations for THM practices and product safety to protect the public from such health risks.

In terms of government support, they perceive a strong commitment and support from the government in regulating THM (73.8%). This finding indicates the need for continued efforts to enhance the comprehensiveness and adequacy of THM legislation, address public health concerns related to THM practices, and ensure effective government support and regulation to protect public health. Furthermore, the study reveals critical information about the knowledge and awareness levels concerning traditional herbal medicine (THM) regulations. Approximately 51.9% of participants lacked sufficient knowledge about THM regulations concerning practices and preparations. The preparation of herbal medicines, particularly related to knowledge, was the primary focus of a report from North Gondar. This report corroborated the findings of the current study, emphasizing the significant role that traditional knowledge plays in the preparation and application of herbal medicines (Asmelashe Gelayee et al., 2017). In the current study, the awareness level regarding herbal medicine regulation was weak for 51.9% of the individuals, and a medium level of awareness was observed in 24.5% of the regulatory personnel. These findings suggest a significant gap in knowledge and awareness, both among the general participants and the regulatory personnel. This indicates a need for improved education and training programs to enhance the understanding and effective implementation of THM regulations.

Regulatory personnel play a critical role in ensuring the quality, safety, efficacy, rational use, and proper storage of traditional herbal medicines (Usure et al., 2024; Demeke et al., 2022). HM effectiveness depends on personnel’s solid awareness and active involvement in the herbal medicine regulatory landscape. In this study, approximately 59.8% of regulatory staff indicated that the rational utilization of THM products available on the market is inadequate. Furthermore, 33.8% of regulatory personnel highlighted that the quality of preparation techniques for HM by licensed traditional herbal medicine practitioners was poor. Additionally, 57.8% of respondents reported that the safety of THM practices was poor. In line with this study, a similar report from Tanzania reported that quality parameters with safety implications were not included in 48% of the active herbal substances (Mssusa et al., 2023). Because the use of herbal treatments continues to raise serious safety concerns, it is essential that the proper regulatory bodies take the necessary steps to safeguard the public’s health by guaranteeing that all herbal medications are both safe and of suitable quality (Ekor, 2014). Furthermore, 33.3% of respondents who work in regulatory offices reported that the storage conditions of HM at home for registered THMPs were inadequate. Properly scientifically sound storage conditions are critical for the shelf life of herbal medicine for safety and quality inheritance. The report from Kenya indicated that lack of acceptance/formal recognition by the relevant authorities, lack of representation, and poor harvesting and storage conditions were the main activities mentioned by practitioners (Okumu et al., 2017). The study presents a critical assessment of the Ethiopian Food and Drug Authority (EFDA) in terms of its performance in regulating traditional herbal medicines (THM).

A significant majority (70.2%) of EFDA regulatory personnel believe that the overall performance of their authority in the practical implementation of HM (herbal medicine) product regulatory activities was weak. Out of a total of 180 RHR respondents, 41.7% of participants reported the weak performance of their regulatory offices in implementing THM regulatory activities. The high percentage of EFDA personnel acknowledging weak regulatory performance suggests significant gaps in the regulatory framework and its enforcement. As the study showed, the weak enforcement of regulatory frameworks could lead to the proliferation of substandard or unsafe herbal and modern pharmaceutical products in the market, posing serious risks to public health (Balkrishna et al., 2024). The EFDA still has flaws in controlling both herbal medicines and modern pharmaceutical products (Suleman et al., 2016).

This study shed light on various aspects of traditional herbal medicine (THM) practices and product regulations, including the existence of monitoring and evaluation systems, self-assessment, work plans, and reporting systems. THM Practices and Product Regulation Plan, Monitoring, and Evaluation. The study participants agreed with the existence of monitoring and evaluation (63.7%), self-assessment (53.6%), work plan (81.9%), and reporting systems (68.4%) for THM regulatory activities. Despite the presence of these systems, 46.41% of respondents report a lack of self-assessment in THM practices, product regulation plans, monitoring, and evaluation. A significant gap exists in self-assessment practices among those involved in traditional herbal medicine (THM) regulations. Encouraging a culture of self-assessment and providing targeted training could help address this issue. By fostering a habit of regular self-evaluation, regulatory personnel can identify areas for improvement and enhance the overall effectiveness of their regulatory frameworks. Training programs could focus on teaching how to conduct thorough self-assessments and how to use the findings to make data-driven improvements in THM practices. This approach could help ensure that regulatory systems are more robust and responsive to emerging challenges (Saggar et al., 2022).

In this study, awareness, reporting trends, and sanctions applied to THM-related problems were surveyed for forwarding regulatory recommendations, which are critical for identifying and reporting issues related to THMs. The study found that unregistered THM services rendering premises and unregistered practitioners were reported by 18.6% (37/199) and 18.6% (40/215) of participants, respectively. More than 50% of the reported THM-related problems received sanctions from regulatory offices. Most study participants noted that legal and administrative sanctions were applied to the reported problems. Most respondents reported the absence of a formal reporting system, a lack of strong legal branches on TM, supposing the problem would not have a severe effect on public health, lack of evidence to report the problems, lack of public support, weak legal enforcement, and educational legal and administrative sanctions not being applied by regulatory offices as reasons for not reporting THM problems. In Ethiopia, a similar issue has been observed where weak legal enforcement contributes to the underreporting of problems related to herbal medicines (Demeke et al., 2022). This issue reflects broader challenges in regulatory frameworks and public health management.

Evaluating the factors that impact the performance of regulatory offices in regulating THM is essential for enhancing the efficiency of these regulatory entities. The study revealed that most respondents identified the lack of research on THM (90.3%), inadequate mechanisms for regulating THM practice and products (87.8%), limited public support (86.5%), weak enforcement of legislation (84.8%), constraints in human and financial resources (84.4%), and insufficient basic regulatory tools for THM (80.6%). It seems that Kenya faces similar challenges in herbal medicine regulation, with weak enforcement of legislation being a primary concern (Okumu et al., 2017). Addressing these concerns may involve increasing funding for research, developing stronger regulatory mechanisms, raising public awareness and support, strengthening legislative frameworks, and improving resource allocation (Ng et al., 2022). These measures would contribute to enhancing the overall effectiveness of regulatory bodies in overseeing THM.

The study also aimed to identify key obstacles associated with the regulation of traditional herbal medicine (THM) products, practices, and practitioners. As per the findings, traditional healers were suspicious of collaborating with scientific communities and showed reluctance to engage with them. The primary challenges confronting THM products included the insufficiency of current inspections conducted through various regulatory approaches (56.5%), the absence of scientific evidence on the safety, effectiveness, and quality of certain medicinal plants (55.3%), and the need for improved procedures and oversight of providers, tasks that fall under the jurisdiction of regional health regulatory bodies and the Ethiopian Food and Drug Authority (EFDA). This urgent need calls for better procedures and oversight of providers, which are within the purview of regional health regulatory bodies and the EFDA. A comparable report emerged from Australia, highlighting the challenges associated with establishing a framework for acquiring the necessary evidence to validate traditional herbal medicinal products, as well as defining a regulatory system that ensures sufficient research funding and safeguards the resulting intellectual property (Roufogalis, 2015). This undermines the broader issue of integrating traditional medicine into regulatory systems while ensuring safety, efficacy, and quality.

## 5 Conclusion and outlook

The study aimed to evaluate the legislative and regulatory framework for the enforcement of THM regulations in Ethiopia. The study revealed deficiencies in the EFDA declaration regarding the definition of TM/HM products and practices; uncertainty surrounding the names, authority, and duties of TM regulatory bodies; lack of clarity on the sources, types, and criteria for regulated TM products; and the necessary connections between TM regulatory entities. The study found that only 34.4% of participants reported that the legislative content on THM was adequate. There was also a lack of knowledge about THM regulations. Additionally, 51.9% of regulatory personnel reported weak herbal medicine practices. Reasons for not reporting THM-related issues included the absence of a formal reporting system, inadequate laws, a lack of evidence, and ineffective legal enforcement. Approximately 70.2% of regulatory personnel expressed that their authority in implementing HM product regulatory activities was insufficient. The main factors impeding regulatory offices in terms of THM regulation were inadequate research on THM (90.3%) and a lack of appropriate mechanisms for regulating THM practices and products (87.8%). Other challenges included healers’ hesitancy to cooperate with scientific communities (56.5%), inadequate THM inspections (55.3%), and a lack of scientific evidence on the safety and effectiveness of medicinal plants (54.4%), which were critical obstacles in regulating THM products, practices, and practitioners.

For improvements to the future roadmap, strengthening the legal framework, enhancing knowledge and capacity, improving research and evidence bases, developing robust reporting and inspection systems, enhancing public support, and legal enforcement are pivotal in the advancement of traditional herbal medicine legislative and regulatory frameworks. By undertaking these critical areas, the EFDA has the capacity to enhance the supervision of traditional herbal medicine products, techniques, and experts, thereby ensuring the safety, quality, and efficacy of herbal medicine-derived products.

## Data Availability

The original contributions presented in the study are included in the article/Supplementary Material; further inquiries can be directed to the corresponding author.
