# Supplementary material for: Traditional herbal medicine legislative and regulatory framework: a cross-sectional quantitative study and archival review perspectives
Source: Front Pharmacol. 2025 Jan 30;16:1475297. doi: 10.3389/fphar.2025.1475297 (PMC11821589; doi:10.3389/fphar.2025.1475297)
Supplement: Supplementary file 1 [file Table1.docx]

**Supplementary File 1.** List of Countries with Existence of Total WHO Used Parameters to Assess Performance of Its Members State on TM Regulation Status

| **Country Name** | **National health policies and national health strategic plans that included TM** | **Exclusive national policy for TM** | **Legal framework for TM** | **Legal and regulation framework for TM practices** | **Code of ethics for TH Practitioners** | **Strategic plan for TM policy implementation** | **National TM programme in Ministry of Health** | **National expert committee for TM** | **National research institutes dedicated for TM or herbal medicines** | **Local production of TM products** | **Regulation systems for HM** | **Registration systems for HM** | **National monographs for he herbal medicine** | **National pharmacopoeia for herbal medicine** | **PMS system for herbal medicine** | **NEML that including TMs products** | **Institutionalized training programmers for TH Practitioners** | **Functional collaboration between THPs and CHPs** | **Frameworks/ policy for protection of IPR/TMK** | **National plan for integrating TM into the national health service delivery** | **Health insurance that includes TM** | **Public and/or government fund for TM** | **Country Total Mark for Y only in %.** |
| --- | --- | --- | --- | --- | --- | --- | --- | --- | --- | --- | --- | --- | --- | --- | --- | --- | --- | --- | --- | --- | --- | --- | --- |
| **Algeria** | U | U | U | U | U | U | Y | U | U | U | U | U | Y | U | U | U | U | U | U | U | U | U | **9.09** |
| **Angola** | U | Y | Y | Y | Y | U | Y | Y | U | U | U | U | Y | U | A | U | A | U | Y | U | A | U | **36.4** |
| **Benin** | U | Y | Y | Y | Y | Y | Y | Y | Y | Y | Y | Y | Y | U | Y | U | Y | U | U | Y | A | Y | **72.7** |
| **Botswana** | U | Y | U | U | U | U | U | U | U | U | U | U | U | U | U | U | U | Y | Y | U | A | U | **13.6** |
| **Burkina Faso** | Y | Y | Y | Y | Y | U | Y | Y | Y | Y | Y | Y | Y | U | U | Y | Y | Y | U | U | A | U | **68.2** |
| **Burundi** | U | Y | Y | Y | U | U | Y | U | U | U | U | U | U | U | U | U | U | U | U | U | A | U | **18.2** |
| **C. African Republic** | U | Y | Y | Y | Y | Y | Y | U | U | U | Y | Y | U | U | U | U | U | Y | U | U | A | U | **40.9** |
| **Cameroon** | Y | Y | Y | Y | Y | U | Y | U | Y | Y | Y | Y | Y | U | U | Y | U | U | Y | U | U | Y | **63.6** |
| **Chad** | Y | Y | Y | Y | Y | Y | Y | Y | Y | U | U | U | Y | U | U | U | U | U | Y | U | U | Y | **54.5** |
| **R. Congo** | Y | Y | U | Y | Y | Y | Y | U | U | Y | U | U | Y | U | U | U | Y | U | U | U | U | U | **40.9** |
| **Côte d’Ivoire** | Y | Y | Y | Y | Y | Y | Y | Y | Y | Y | Y | Y | Y | Y | Y | Y | Y | U | U | U | U | U | **77.3** |
| **DR of the Congo** | Y | Y | Y | Y | Y | Y | Y | Y | Y | Y | Y | Y | Y | Y | Y | Y | U | U | U | U | U | Y | **77.3** |
| **Egypt** | Y | U | Y | Y | Y | Y | Y | Y | U | U | U | U | Y | Y | Y | U | U | U | U | U | U | Y | **50** |
| **Equatorial Guinea** | U | Y | Y | Y | Y | U | Y | U | Y | U | U | Y | Y | U | U | U | U | U | U | U | U | Y | **40.9** |
| **Eritrea** | U | A | A | Y | U | U | Y | U | U | U | U | U | U | U | U | U | U | U | Y | U | U | Y | **18.2** |
| **Ethiopia** | Y | Y | Y | Y | U | Y | Y | U | Y | U | Y | Y | U | U | U | U | U | Y | U | U | U | Y | **50** |
| **Gabon** | Y | Y | Y | Y | U | Y | Y | U | Y | U | U | U | U | U | U | U | U | U | U | U | U | Y | **36.4** |
| **Gambia** | Y | Y | Y | U | U | Y | Y | Y | U | U | U | U | Y | U | U | U | U | U | Y | U | U | U | **36.4** |
| **Ghana** | Y | Y | Y | Y | Y | Y | Y | Y | Y | Y | Y | Y | Y | Y | Y | Y | Y | U | Y | Y | U | Y | **90.9** |
| **Guinea** | Y | Y | Y | Y | Y | Y | Y | U | Y | Y | Y | U | Y | Y | U | U | U | U | U | U | U | U | **54.5** |
| **Guinea-Bissau** | U | Y | Y | Y | Y | Y | Y | U | U | U | U | U | U | U | U | U | U | U | U | U | U | U | **27.3** |
| **Kenya** | Y | U | U | A | U | U | Y | U | U | U | U | U | U | U | U | U | U | U | Y | U | U | U | **13.6** |
| **Lesotho** | U | U | U | Y | U | U | U | U | U | U | U | U | U | U | U | U | U | U | U | U | U | U | **4.55** |
| **Liberia** | U | Y | Y | Y | U | U | Y | U | U | U | U | U | U | U | U | U | U | U | U | U | U | U | **18.2** |
| **Madagascar** | Y | Y | Y | Y | U | Y | Y | Y | Y | Y | Y | Y | Y | Y | Y | Y | Y | U | U | U | U | Y | **77.3** |
| **Malawi** | Y | Y | Y | Y | U | Y | Y | U | U | U | Y | U | U | U | U | U | U | Y | Y | U | U | U | **40.9** |
| **Mali** | Y | Y | Y | Y | Y | Y | Y | Y | Y | Y | U | Y | Y | U | Y | Y | Y | Y | Y | Y | Y | Y | **90.9** |
| **Mauritania** | Y | Y | Y | Y | Y | Y | Y | Y | U | U | U | U | U | U | Y | U | U | U | U | U | U | U | **40.9** |
| **Morocco** | U | U | Y | U | U | U | Y | U | U | U | U | U | Y | U | Y | U | U | U | U | U | U | U | **18.2** |
| **Mozambique** | Y | Y | Y | Y | U | Y | Y | Y | Y | Y | Y | Y | Y | U | Y | U | Y | Y | Y | U | U | Y | **77.3** |
| **Namibia** | Y | Y | Y | Y | U | U | U | U | U | U | U | Y | U | U | Y | U | U | U | U | U | U | U | **27.3** |
| **Niger** | Y | Y | Y | U | U | U | Y | U | U | U | Y | Y | Y | U | U | Y | Y | Y | U | U | U | Y | **63.6** |
| **Nigeria** | Y | Y | Y | Y | Y | Y | Y | Y | Y | Y | Y | Y | Y | Y | Y | U | Y | Y | Y | U | U | Y | **86.4** |
| **Rwanda** | Y | Y | Y | Y | Y | Y | Y | U | Y | Y | U | U | Y | U | U | U | U | U | U | U | U | U | **45.5** |
| **Sao Tome and Principe** | U | Y | U | U | U | Y | Y | U | U | U | U | U | U | U | U | U | U | U | U | U | U | U | **13.6** |
| **Senegal** | U | Y | Y | Y | Y | Y | Y | Y | Y | Y | Y | U | Y | U | U | U | Y | Y | U | U | U | U | **59.1** |
| **Sierra Leone** | U | Y | Y | Y | U | U | Y | Y | U | U | Y | Y | U | U | U | U | U | U | U | U | U | U | **31.8** |
| **Somalia** | U | U | U | Y | A | U | U | U | U | U | U | U | U | U | U | U | U | U | U | U | U | U | **4.55** |
| **South Africa** | Y | Y | Y | Y | Y | Y | Y | Y | Y | Y | Y | Y | Y | Y | Y | U | Y | Y | Y | U | Y | Y | **90.9** |
| **South Sudan** | U | U | U | U | A | A | Y | U | U | U | U | U | U | U | U | U | U | U | U | U | U | U | **4.55** |
| **Sudan** | Y | Y | Y | Y | U | U | Y | U | U | U | U | U | Y | U | U | U | U | U | U | U | U | Y | **31.8** |
| **Togo** | Y | Y | Y | Y | U | U | Y | U | Y | Y | Y | U | Y | U | U | U | U | U | Y | U | U | U | **45.5** |
| **Tunisia** | U | U | Y | Y | U | U | Y | U | U | U | U | U | Y | U | U | Y | U | U | U | U | U | Y | **27.3** |
| **Uganda** | U | Y | Y | Y | Y | Y | Y | Y | Y | Y | Y | Y | Y | Y | Y | U | Y | Y | U | U | U | Y | **77.3** |
| **UR of Tanzania** | Y | Y | Y | Y | Y | Y | Y | Y | Y | Y | Y | Y | U | U | U | U | Y | Y | U | U | U | Y | **68.2** |
| **Zambia** | Y | Y | Y | Y | Y | Y | Y | Y | Y | U | U | Y | U | U | U | U | U | Y | U | U | U | U | **50** |
| **Zimbabwe** | Y | Y | Y | Y | Y | Y | Y | U | Y | U | U | Y | U | U | Y | U | U | Y | Y | U | U | Y | **59.1** |
| **China** | Y | Y | Y | Y | Y | Y | Y | Y | Y | Y | Y | Y | Y | Y | Y | Y | Y | Y | Y | Y | Y | Y | **100** |
| **India** | Y | Y | Y | Y | Y | Y | Y | Y | Y | Y | Y | Y | Y | Y | Y | Y | Y | Y | Y | Y | Y | Y | **100** |

***Note:-*** **U=** *Information not Available***, A=** *Absent***, Y=** *Present*
