# Supplementary material for: Traditional herbal medicine legislative and regulatory framework: a cross-sectional quantitative study and archival review perspectives
Source: Front Pharmacol. 2025 Jan 30;16:1475297. doi: 10.3389/fphar.2025.1475297 (PMC11821589; doi:10.3389/fphar.2025.1475297)
Supplement: Supplementary file 2 [file Table2.docx]

## Supplementary File 2. List of selected medicine and health regulatory bodies for the study

| **Regulatory Level of Selected Organizations** | **Name of Selected Medicine and Health Authorities or Institutions or Offices** |
| --- | --- |
| **Federal level** | Product assessment and registration directorate under EFDA |
|  | Licensing and inspection directorate under EFDA |
|  | Product safety directorate under EFDA |
|  | Product quality assessment directorate under EFDA |
|  | Central, Veterinary Drugs and Animal Feed Administration and Control Authority (VDAFACA) |
|  | Ethiopia public health institute (EPHI) |
|  | Pharmaceutical and medical equipment administration directorate under MoH |
| **Regional level** | Oromia regional state, Health and Health Related Services and Product Quality Control Directorate (HHRSPQCD) |
|  | SNNPR regional state, Health and Health Related Services and Product Quality Control Authority (HHRSPQCA) |
|  | Addis Ababa city Food, Health, Healthcare Administration and Control Authority (FMHACA) |
| **Zonal level (from Oromia and SNNPR Regional States)** | East Shewa zona health and health related service and product quality control offices |
|  | East wollega zona health and health related service and product quality control offices |
|  | Jimma zona health and health related service and product quality control offices |
|  | West Arsi zona health and health related service and product quality control offices |
|  | Ilu-Ababor zona health and health related service and product quality control offices |
|  | Bale zona health and health related service and product quality control offices |
|  | Wast Hararghe zona health and health related service and product quality control offices |
|  | Gedio zona health and health related service and product quality control offices |
|  | Wolitia zona health and health related service and product quality control offices |
|  | Gurage zona health and health related service and product quality control offices |
|  | Gamo zona health and health related service and product quality control offices |
|  | Hadiya zona health and health related service and product quality control offices |
| **Sub-cities level (Addis Ababa city)** | Bole sub-city food, health, healthcare administration and control offices |
|  | Akaki-Kalit sub-city food, health, healthcare administration and control offices |
|  | Addis-Katam sub-city food, health, healthcare administration and control offices |
|  | Kikos sub-city food, health, healthcare administration and control offices |
| **Zonal Town level (Oromia and SNNPR regions)** | Adama town health and health related service and product quality control office |
|  | Jimma town health and health related service and product quality control office |
|  | Nekemte town health and health related service and product quality control office |
|  | Robe town health and health related service and product quality control office |
|  | Mattu town health and health related service and product quality control office |
|  | Shashamane town health and health related service and product quality control office |
|  | Bushoftu town health and health related service and product quality control office |
|  | Batu town health and health related service and product quality control office |
|  | Ciro town health and health related service and product quality control office |
|  | Dila town health and health related service and product quality control office |
|  | Hosaena town health and health related service and product quality control office |
|  | Walkite town health and health related service and product quality control office |
|  | Arba-Minche town health and health related service and product quality control office |
|  | Sodo town health and health related service and product quality control office |
| **Woreda level (Addis Ababa city)** | At Bole sub-city, Woreda 01, 04, 05, 07 and 12; food, health, health care administration and control offices |
|  | At Akaki-Kalit sub-city; Woreda 03, 06 and 08; food, health, healthcare administration and control offices |
|  | At Addis-Katam sub-city; Woreda 02, 05, 07 and 09; food, health, healthcare administration and control offices |
|  | At Kikos sub-city; Woreda 08 and 10; food, health, healthcare administration and control offices |
