# Supplementary material for: Traditional herbal medicine legislative and regulatory framework: a cross-sectional quantitative study and archival review perspectives
Source: Front Pharmacol. 2025 Jan 30;16:1475297. doi: 10.3389/fphar.2025.1475297 (PMC11821589; doi:10.3389/fphar.2025.1475297)
Supplement: Supplementary file 3 [file Table5.docx]

**Supplementary File 5. Awareness, reporting trend and applied sanction of THM related problems, Ethiopia, 2022 (n=237)**

| Variables | Aware of THM related problems | | | Reported THM problems | | Sanction applied on reported problems | |
| --- | --- | --- | --- | --- | --- | --- | --- |
|  |  | n | % | n | %* | n | %** |
| Unregistered THM services rendering establishments | Yes | 199 | 84.0 | 37 | 18.6 | 26 | 70.3 |
|  | No | 38 | 16.0 | 162 | 81.4 | 11 | 29.7 |
| Unregistered THM Practitioners | Yes | 215 | 90.7 | 40 | 18.6 | 25 | 62.5 |
|  | No | 22 | 9.3 | 175 | 81.4 | 15 | 37.5 |
| Unregistered THM practices | Yes | 191 | 80.6 | 35 | 18.3 | 23 | 65.7 |
|  | No | 46 | 19.4 | 156 | 81.7 | 12 | 34.3 |
| Poor quality, safe and efficacy of HM preparation/products | Yes | 203 | 85.7 | 39 | 19.2 | 23 | 59.0 |
|  | No | 34 | 14.3 | 164 | 80.8 | 16 | 41.0 |
| THM practices safety problems | Yes | 133 | 56.1 | 28 | 21.1 | 17 | 60.7 |
|  | No | 104 | 43.9 | 105 | 78.9 | 11 | 39.3 |
| Problem related to price affordability | Yes | 82 | 34.6 | 3 | 3.7 | 0 | 0.0 |
|  | No | 155 | 65.4 | 79 | 96.3 | 3 | 100.0 |
| Misleading/in-accurate THM promotion and advertising | Yes | 170 | 71.7 | 29 | 17.1 | 19 | 65.5 |
|  | No | 67 | 28.3 | 141 | 82.9 | 10 | 34.5 |
| THM marketing related problems | Yes | 175 | 73.8 | 35 | 20.0 | 24 | 68.6 |
|  | No | 62 | 26.2 | 140 | 80.0 | 11 | 31.4 |

**Note:** *Percentage of total aware or “Yes” for awareness ** Percentage of total reported problems or “Yes” for reported THM problems
