# Supplementary material for: Traditional herbal medicine legislative and regulatory framework: a cross-sectional quantitative study and archival review perspectives
Source: Front Pharmacol. 2025 Jan 30;16:1475297. doi: 10.3389/fphar.2025.1475297 (PMC11821589; doi:10.3389/fphar.2025.1475297)
Supplement: Supplementary file 4 [file Table3.docx]

## Supplementary File 3. List of Countries with Existence of Total Key Futures Used in Checklist

| **Country Name** | **Exist Parameters** | **Absent or Information not Available Parameters** | **Total Parameters in Present** |
| --- | --- | --- | --- |
| 1. China | 22 | 0 | **100** |
| 1. India | 22 | 0 | **100** |
| 1. Ghana | 20 | 2 | **90.90** |
| 1. Mali | 20 | 2 | **90.90** |
| 1. South Africa | 20 | 2 | **90.90** |
| 1. Nigeria | 19 | 3 | **86.36** |
| 1. Côte d’Ivoire | 17 | 5 | **77.27** |
| 1. D. Republic of Congo | 17 | 5 | **77.27** |
| 1. Madagascar | 17 | 5 | **77.27** |
| 1. Mozambique | 17 | 5 | **77.27** |
| 1. Uganda | 17 | 5 | **77.27** |
| 1. Benin | 16 | 6 | **77.27** |
| 1. Burkina Faso | 15 | 7 | **68.18** |
| 1. UR of Tanzania | 15 | 7 | **68.18** |
| 1. Cameroon | 14 | 8 | **63.63** |
| 1. Niger | 14 | 8 | **63.63** |
| 1. Senegal | 13 | 9 | **59.09** |
| 1. Zimbabwe | 13 | 9 | **59.09** |
| 1. Chad | 12 | 10 | **54.54** |
| 1. Guinea | 12 | 10 | **54.54** |
| 1. Egypt | 11 | 11 | **50** |
| 1. Ethiopia | 11 | 11 | **50** |
| 1. Zambia | 11 | 11 | **50** |
| 1. Rwanda | 10 | 12 | **45.45** |
| 1. Togo | 10 | 12 | **45.45** |
| 1. Central of African Republic | 9 | 13 | **40.90** |
| 1. Republic of Congo | 9 | 13 | **40.90** |
| 1. Equatorial Guinea | 9 | 13 | **40.90** |
| 1. Malawi | 9 | 13 | **40.90** |
| 1. Mauritania | 9 | 13 | **40.90** |
| 1. Angola | 8 | 16 | **36.36** |
| 1. Gabon | 8 | 14 | **36.36** |
| 1. Gambia | 8 | 14 | **36.36** |
| 1. Sierra Leone | 7 | 15 | **31.81** |
| 1. Sudan | 7 | 15 | **31.81** |
| 1. Guinea Bissau | 6 | 16 | **27.27** |
| 1. Namibia | 6 | 16 | **27.27** |
| 1. Tunisia | 6 | 16 | **27.27** |
| 1. Burundi | 4 | 18 | **18.18** |
| 1. Eritrea | 4 | 18 | **18.18** |
| 1. Liberia | 4 | 18 | **18.18** |
| 1. Morocco | 4 | 18 | **18.18** |
| 1. Botswana | 3 | 19 | **13.63** |
| 1. Kenya | 3 | 19 | **13.63** |
| 1. Sao Tome and Principe | 3 | 19 | **13.63** |
| 1. Algeria | 2 | 20 | **9.09** |
| 1. Lesotho | 1 | 21 | **4.54** |
| 1. Somalia | 1 | 21 | **4.54** |
| 1. South Sudan | 1 | 21 | **4.54** |
