# Supplementary material for: Traditional herbal medicine legislative and regulatory framework: a cross-sectional quantitative study and archival review perspectives
Source: Front Pharmacol. 2025 Jan 30;16:1475297. doi: 10.3389/fphar.2025.1475297 (PMC11821589; doi:10.3389/fphar.2025.1475297)
Supplement: Supplementary file 5 [file Table4.docx]

**Supplementary File 4. Demographic characteristics of regulatory personnel, Ethiopia, 2022 G.C, (n=237).**

| Respondents personal Information | | | Freq. (n) | Percent (%) |
| --- | --- | --- | --- | --- |
| Gender | | Male | 179 | 75.5 |
|  |  | Female | 58 | 24.5 |
| Profession | | Pharmacist | 157 | 66.2 |
|  |  | Environmental Health | 24 | 10.1 |
|  |  | Health Officer | 37 | 15.6 |
|  |  | Nursing | 15 | 6.3 |
|  |  | Medical Laboratory | 4 | 1.7 |
| Education level | | Diploma | 4 | 1.7 |
|  |  | Degree | 197 | 83.1 |
|  |  | Master (MSc) | 36 | 15.2 |
| Regulatory authority and offices | Federal | FDA of Ethiopia | 57 | 24.1 |
|  | Oromia region | Oromia region | 15 | 6.3 |
|  |  | Oromia zone/town offices | 43 | 18.1 |
|  | SNNPR region | SNNPR regional office | 16 | 6.8 |
|  |  | SNNPR zone/town offices | 25 | 10.5 |
|  | Addis Ababa city | Addis Ababa city FMHACA | 11 | 4.6 |
|  |  | Sub-cities FMHAC offices | 13 | 5.5 |
|  |  | Woredas’ FMHAC offices | 57 | 24.1 |
| Respondent regulation experience | | ≤ 4 year | 139 | 58.6 |
|  |  | > 4 years | 98 | 41.4 |
